# PReS-FINAL-2220: Cochlear involvement in FMF

**DOI:** 10.1186/1546-0096-11-S2-P210

**Published:** 2013-12-05

**Authors:** G Keskindemirci, N Aktay Ayaz, A Batioglu, G Aydogan, E Aldemir, Z Dönmez, Ö Yiğit, S Ozen

**Affiliations:** 1 Kanuni Sultan Suleyman Education and Research Hospital, Istanbul, Turkey; 2 Istanbul Education and Research Hospital, Istanbul, Turkey; 3 Hacettepe Medical Faculty, Ankara, Turkey

## Introduction

FMF is a monogenic autoinflammatory disease with recurring episodes of fever and serositis attacks. FMF is associated with mutations in pyrin. On the other hand mutations in a molecule in the same pathway, cryopyrin, is characterized by inflammatory features involving the inner ear as well. A study has suggested the involvement of cochlea in Behçet disease, which is a polygenic autoinflamatory disease.

## Objectives

To evaluate the cochlear function of children with the diagnosis of FMF prospectively.

## Methods

Children included to the study were diagnosed as FMF according to previously suggested criteria. 74 children with FMF and 20 controls were enrolled to the study. Demographic data and MEFV mutation analysis were recorded. Patients with any middle and external ear pathology were excluded from the study. After otoscopic inspection, audiometric examinations were carried out including otoacustic emission testing (OAE) by distortion products (DP) and signal noise ratio (SNR) testing with 1000, 1400, 2000, 2800 and 4000 Hz and audiometric evalution with pure tone average (PTA) measurements and high frequency levels of 8000, 10000, 12500, 16000 Hz.

## Results

All patients had genetic analysis for the most common mutations of our country (M694V, R202Q, E148Q, M680I, V726A, K695R). The patient group included 74 children (46 female and 28 male patient) with mean age 10.9 (range 26 months- 18 years) and the control group was age and sex matched. PTA levels were normal in both FMF patients and the control group. However, hearing levels at the frequency of 10000 Hz, 12500 Hz and 16000 Hz were found to be significantly higher in the FMF. In OAE tests, DP and SNR of FMF group was lower at 1000 Hz frequency.

## Conclusion

The most common mutations were M694V, R202Q, E148Q, M680I, V726A, K695R. There was no correlatation between the genetic mutations and cochlear involvement. Cochlear function is very important for learning in the childhood. Like in other cryopyrinopathies, cochleovestibular involvement in FMF is probable and this may be attribueted **to subclinical inflamation **present during the disease course. Further studies are needed to understand whether these subtle changes are significant.

## Disclosure of interest

None declared.

